# Supplementary material for: Multilingual capabilities of GPT: A study of structural ambiguity
Source: PLoS One. 2025 Jul 7;20(7):e0326943. doi: 10.1371/journal.pone.0326943 (PMC12233270; doi:10.1371/journal.pone.0326943)
Supplement: S1 Table — (PDF) [file pone.0326943.s008.pdf]

The proportion of LA preferences by Claude 3.7 Sonnet

**Claude 3.7 sonnet**	**All types**	**60.71% (765/1260)****	**76.11% (959/1260)****	**64.04% (807/1260)***
	Question type A	58.57% (246/420)	76.90% (323/420)**	66.19% (278/420)**
	Question type B	64.28% (270/420)	69.52% (292/420)**	59.28% (249/420)
	Question type C	68.80% (289/420)**	81.90% (344/420)**	66.66% (280/420)**

Asterisks (**) indicate significant differences and (*) indicate marginal differences between LA and HA interpretations (‘**’ indicates $p < 0.05$ and ‘*’ indicates a p-value between 0.05 and 0.10).
