# Supplementary material for: Multilingual capabilities of GPT: A study of structural ambiguity
Source: PLoS One. 2025 Jul 7;20(7):e0326943. doi: 10.1371/journal.pone.0326943 (PMC12233270; doi:10.1371/journal.pone.0326943)
Supplement: Attachment — Submitted filename: Response to Reviewers.pdf [file pone.0326943.s010.pdf]

Editor's Comments

- As you can see from the attached, while the reviewers comment that your study has the potential to contribute significantly to the literature, they have raised several theoretical and methodological issues with it. Reviewer 1 is more enthusiastic than Reviewer 2. I also read your manuscript carefully. I agree with both reviewers that the manuscript is interesting. I also share the main concern of Reviewer 2 about the fact that as you have focused on a single version of GPT, you need to widen the scope of the manuscript.

  Response: We sincerely appreciate the constructive and thoughtful feedback and share the view that broadening the scope of model coverage can enhance the depth and generalizability of the study. We fully acknowledge that a more extensive comparison across fundamentally different large language model architectures would be valuable and informative. However, as noted by Reviewer 2, conducting such a comparative analysis would require a significantly different approach and framing throughout the paper. Thus, after careful consideration and in line with Reviewer 2's suggestion, we expanded our investigation to include two of OpenAI's recent models—o1-mini (September 2024) and o3-mini (January 2025)—which have not yet been systematically explored in the context of scope ambiguity.

  While these models are part of the GPT family, they differ meaningfully from earlier versions in terms of their architecture, including tuning strategies, optimization goals, and model scales. As such, we believe this approach enriches the manuscript by offering broader implications of our findings that can guide improvements in language model performance in future developments while maintaining a coherent and focused scope for the current study. We deepen the discussion by engaging in more fine-grained model comparisons through the inclusion of two newer, underexplored models.

  Additionally, in the Discussion section, we briefly report findings from an exploratory evaluation using Anthropic's Claude 3.5 Sonnet, which similarly showed LA preferences across all three languages. These results are provided in the Supporting Information and included as part of our suggestions for future research.

  To further reflect this expansion and strengthen the overall contribution of our paper, we have highlighted several key points in the revised manuscript:

  (1) ***Abstract****: Updated to reflect the inclusion of findings from o1-mini and o3-mini to provide a more complete overview of the study's contributions (lines 9-13).*
  (2) ***Introduction****: clarified the rationale for focusing on GPT models and emphasize the significance of comparing different model versions.*
  (3) ***Materials and Methods****: Added detailed descriptions of the architectural and functional differences across all GPT models examined, including o1-mini and o3-mini.*
  (4) ***Analysis and Results****: Integrated new results from o1-mini and o3-mini, with proportions and statistical analysis presented in Table 2.*
  (5) ***Discussion****: divided into two subsections—Cross-linguistic Model Comparisons and Model Reasoning and Future Directions— to improve readability and better highlight the implications of our findings through more in-depth discussion.*
  (6) ***Supporting Information****: Included the dataset and results from an exploratory evaluation using Anthropic's Claude 3.5 Sonnet, which showed similar LA preferences across languages, to support the generalizability of our findings and guide future work.*

  We hope these revisions broaden the manuscript's scope while maintaining a focused and coherent contribution. The details of each revision are provided below:

1. *To clarify the rationale for investigating GPT models, we have added a statement in the Introduction highlighting their dominant role in both academic research and real-world applications (lines 20-24). We emphasized that GPT's widespread deployment makes it a natural and impactful starting point for investigating how large language models (LLMs) handle complex linguistic tasks. Additionally, we now include a statement outlining the significance of comparing multiple GPT versions—including the recent o1-mini and o3-mini models—to examine how underlying techniques and model scale shape interpretive behavior across languages (lines 82-88). This comparison, we argue, is essential for guiding the practical implications of GPT model selection and systematic strategies for enhancing multilingual capabilities.*

2. *In Materials and methods section, we now detail characteristics of each GPT model, including the recently released o1-mini and o3-mini models (lines 267-284). In particular, we highlight the inclusion of o1-mini and o3-mini as an important contribution, given their underexplored performance on complex linguistic phenomena, including structural ambiguity, and their distinct technical characteristics relative to other GPT models. We believe this addition allows for a more comprehensive analysis of interpretive behavior across different stages of GPT development.*

3. *The results of o1-mini and o3-mini are now included in Table 2 (pp. 15-17) and integrated into the Analysis and Results section (lines 318-356). Notably, these models showed strong LA preferences in both Japanese and Korean—mirroring the pattern observed in English. This contrasts with earlier GPT models, in which Japanese did not exhibit a strong preference for either interpretation, and GPT-4o even showed a significant HA preference, aligning more closely with native Japanese speakers. These findings from o1-mini and o3-mini are now incorporated into the manuscript to support a deeper discussion of model behavior across different versions.*

4. *We have extended the discussion to provide a more detailed comparison of GPT models, focusing on their distinct architectures and alignment strategies. As a result of this expanded analysis, the Discussion section is now divided into two subsections—(1) Cross-linguistic Model Comparisons and (2) Model Reasoning and Future Directions—to enhance readability and clearly present these key points. This restructuring reflects our deeper exploration of GPT model comparisons and allows us to systematically examine how differences in model design, scale, and training influence interpretive behavior across languages. These detailed comparisons are now included in the Cross-linguistic Model Comparisons subsection (lines 391-394; lines 403-431).*

   Additionally, in the Model Reasoning and Future Directions subsection, we briefly report the results of an exploratory evaluation using Anthropic's Claude 3.5 Sonnet, which showed a similar pattern of strong LA preferences across all three languages. Since it goes beyond the scope of the current study, these findings are provided in the Supporting Information to support our discussion of broader interpretive tendencies across LLMs and to inform directions for future cross-architecture comparisons (lines 477-488).

- Line 76, there is a typo: GPT mdoels′ instead of GPT models

Response: We have corrected the errors – thank you for noting this (line 33)

- Lines 100 to 105. You may want to also include European and Brazilian Portuguese:

Soares, A. P., Fraga, I., Comesaña, M., & Piñeiro, A. (2010). El papel de la animacidad en la desambiguación de oraciones de relativo en portugués europeo: evidencia en tareas de producción y comprensión. Psichotema, 22(4), 691-696.

Soares, A. P., Oliveira, H., Ferreira, M., Comesaña, M., Macedo, F., Ferré, P., Acuña-Fariña, J. C., Hernández Cabrera, J., & Fraga, I. (2019). Lexico-syntactic interactions during the processing of temporally ambiguous L2 relative clauses: An eye-tracking study with intermediate and advanced Portuguese-English bilinguals. Plos One, 14(5): e0216779. DOI: 10.1371/journal.pone.0216779

Maia, M., Fernández, E. M., Costa, A., & do Carmo Lourenço-Gomes, M. (2007). Early and late preferences in relative clause attachment in Portuguese and Spanish. Journal of Portuguese Linguistics, 6(1).

Response: We appreciate this suggestion to include European and Brazilian Portuguese. We now note that high attachment (HA) is also preferred by speakers of Brazilian and European Portuguese, citing the recommended studies. We have also added Romanian (Ehrlich et al., 1999) to the list of languages showing low attachment (LA) preferences (lines 105-111).

- Lines 122 to 125. There are more studies that should be included here to soften this statement. See for instance

Acuña-Fariña, C., Fraga, I., García-Orza, J., & Piñeiro, A. (2009). Animacy in the adjunction of Spanish RCs to complex NPs. European Journal of Cognitive Psychology, 21(8), 1137-1165.

In this study, the authors showed a clear HA strategy that is reversed as a function of animacy. I mean, there are several studies using online measures in which the HA preference has been observed.

Response: We appreciate this suggestion to provide a more balanced account of findings from real-time processing studies. In response, we revised the opening sentence of the paragraph to avoid overgeneralization and updated the remainder of the paragraph to more accurately reflect the range of results reported in the literature. Specifically, we replaced the original phrasing with: "While interpretation preferences are often assessed through offline tasks that require judgments after reading entire sentences, psycholinguistic research has examined interpretation preferences in real-time processing to assess initial interpretations.(lines 128-130)" This revised sentence is followed by an introduction of cross-linguistic variability, including Spanish and Japanese as cases where initial low attachment preferences shift toward high attachment in offline interpretation. We also acknowledge the study suggested by the reviewer—Acuña-Fariña et al. (2009)—which demonstrates that early attachment preferences in Spanish can be modulated by linguistic factors such as animacy.(lines 131-143)

- Lines 169 to 172. You should soften this statement considering my previous comments. Also, a brief explanation of why Korean show a different trend than Japanese would be appreciated.

Response: We have softened the original claim to specify that Spanish speakers tend to prefer LA interpretations during initial processing only in the absence of additional semantic or linguistic factors. The revised sentence now reads: "Recall that Japanese speakers initially prefer LA during online processing but shift toward HA in offline tasks, mirroring Spanish speakers when linguistic factors such as animacy or syntactic complexity are not involved. Conversely, Korean speakers consistently maintain an HA interpretation from the initial stages of parsing through to their final interpretations. (lines 184-188)"

Following the statement, we have added a brief explanation suggesting that the divergence between Japanese and Korean, despite their syntactic similarities, may stem from different parsing strategies. Specifically, we propose that Korean speakers' consistent HA preference may reflect greater sensitivity to syntactic cues such as predicate proximity (Gibson et al., 1996), which promotes high attachment early in processing (Lee & Kweon, 2004; Lim, 2012). In contrast, Japanese speakers appear to rely more heavily on locality-based parsing strategies, attaching the relative clause to the closest available noun phrase, resulting in early LA interpretations that later shift toward HA preferences (Kamide & Mitchell, 1997). Please see lines 188-195 in the revised manuscript.

- Lines 185 to 187. If the terms were used for the low attached nouns, how sister can be in NP1? I lost the point here, please clarify it.

  Response: Thank you for pointing out this error. The original sentence mistakenly referred to "low" attached nouns, which caused the confusion. We have corrected this in the revised manuscript to accurately refer to "high" attached nouns (line 210).

- Line 227. There is another typo. An extra dot after "one"

  Response: Thank you for noting this. This has been removed (line 254).

Reviewer 1's Comments

- 1.- Page 5, lines 76 to 83. I think the whole paragraph is out of place, as it belongs more in the discussion or conclusions than in an introduction. The easiest thing to do would be to delete it.

  Response: We thank the reviewer for this suggestion. In response, we have removed the paragraph.

- 2.- On page 12, Materials and methods, it says that three different versions of the GPT were used. It would be good to give a reason for this, or at least what is different/interesting about the three versions used.

  Response: We thank the reviewer for highlighting the need to clarify the rationale behind the selection of GPT models. In the revised manuscript, we have expanded the Materials and Methods section (lines 267–284) to provide a clearer explanation of the characteristics and distinctions among the GPT models included in our analysis. In addition to the originally described models, we evaluated two recently released versions—o1-mini and o3-mini—which are optimized for efficiency and rapid inference.

These models have now been incorporated into this section to reflect their inclusion in our experimental analysis. Their evaluation allows us to explore how differences in model architecture, tuning techniques, and training scale influence syntactic interpretation patterns. We further discuss these differences and their implications in the Discussion section (lines 391-394, lines 409–431).

- 3.- While the results for English are very clear and consistent, question type B in GPT - 4 turbo was not significant, while question type B for GPT -3.5 turbo, with a lower percentage of LA responses, was. Any idea what this might be due to?

  Response: To address this point, we have revised the manuscript to include a possible explanation for the discrepancy in statistical significance. Specifically, we note that GPT-4 turbo showed greater variability depending on which noun phrase was queried. When the model was explicitly asked whether NP1 was modified, it selected NP2 in over 90% of cases; conversely, when NP2 was queried, it selected NP1 over 65% of the time. This reversal pattern suggests that GPT-4 turbo's attachment decisions were more sensitive to the form of the question in type B trials, likely contributing to the lack of statistical significance despite a general LA preference. Supporting this account, statistical analysis revealed that when the random intercept for question type was excluded, the overall LA preference in GPT-4 turbo reached significance. We also discuss potential explanations for the increased variability observed in Question Type B compared to the other question types. (lines 318-333).

  While clarifying this point, we also realized the need to explicitly describe how answer choices and questioned nouns were balanced across different question types. In the previous manuscript, this methodological detail was insufficiently explained. Question types A and C differ only in the order of answer choices presented, while question type B varies in terms of the specific NP queried in the question itself. This clarification regarding the balancing of questions and answer choices has been added to the Materials and methods section (lines 234–238).

- 4.- Pages 18-19, lines 338-350. The GPT models were asked to give an explanation for each answer choice. According to the text, even when the preferred option was HA, the models gave the explanation that 'relative clauses typically modify the nearest preceding noun'. This is interpreted to mean that their reasoning process is not completely language independent. However, I see another possible explanation here: GPT may not be answering on the basis of the criteria it actually used to make the decision, because it is unaware of them. In other words, its answer and the justification could be independent of each other, since it seems to generate explanations uniformly based on a general knowledge of how these ambiguities are usually resolved, rather than on the specific case of the item presented. If this is the case, this part of the discussion would not provide much information about its actual reasoning. I would like to hear about this possibility.

  Response: We thank the reviewer for this insightful observation. We have revised the relevant discussion section to incorporate this alternative interpretation. Specifically, we now acknowledge that GPT's provided rationales may not reflect the actual decision-making criteria it uses during attachment resolution, but may instead function as post-hoc justifications generated independently of the model's internal selection process. We note that this interpretation is consistent with recent concerns raised by Mahowald et al. (2024), who caution against conflating the linguistic fluency of large language models with genuine reasoning ability. Accordingly, we advise caution when interpreting GPT's justifications as direct insight into its internal processing. We also highlight this as an area for future investigation. Please see lines 447-460 in the revised manuscript.

- Typos:

  -Page 3, line 26: mdoels’ -> models’

  Response: This has been corrected. (line 33)

  -Page 5, line 85: The abbreviation RC here is the first time it appears, and it is not used again in the whole article. So, it is better to replace it with the full wording: relative clause.

  Response: Thank you for pointing this out. In accordance with the suggestion, we have replaced the abbreviation "RC" with the full term "relative clause" (line 91).

  -Page 6, line 107: HA preferences). -> remove)

  Response: The extraneous parenthesis has been removed (line 113).

  -Page 8, line 142: Indicate what the acronym LSTM stands for.

  Response: We have now defined the acronym “LSTM” as “Long short-term memory” (lines 156-157).

  -Page 10, line 169: multiliguality -> multilinguality.

  Response: This has been corrected. (line 184)

  -Page 11, lines 205-207: rephrase the sentence.

  Response: Thank you for pointing this out. The original sentence on page 11, lines 205–207 was incomplete and has now been revised for clarity and completeness. The updated sentence reads: “Previous studies have highlighted that LLMs are sensitive to question formatting, particularly in multiple-choice settings, where their responses can be influenced by the structure of the prompt and the order in which answer choices are presented.” (lines 229-231)

  -Page 12, line: 227: remove the full stop at the end.

  Response: This has been corrected. (line 254)

  -Page 13, line 239: different reference format!

  Response: The reference format on page 13, line 239 has been corrected to match the numbering style used throughout the manuscript, and now appears as [35] (line 265).

  -Figure 1. Please, increase figure quality.

  Response: The quality of Figure 1 has been enhanced in the revised manuscript to ensure improved clarity and resolution.

Reviewer 2’s Comments

- The topic of structural ambiguity in large language models is timely and interesting. However, the current version is too focused on GPT, which is continuously evolving, meaning the present patterns may or may not replicate in 2025 versions or newer. As acknowledged by the authors, the study would need to be extended to other large language models, which may have been trained differently.

Perhaps, adding other current models (or even different versions of the same model, such as GPT- 4.0 with o1 or o3) could help the manuscript cross the publication boundary at PLOS One. However, this would require rethinking the overall approach.

Response: We sincerely appreciate the constructive and thoughtful feedback and share the view that broadening the scope of model coverage can enhance the depth and generalizability of the study. We fully acknowledge that a more extensive comparison across fundamentally different large language model architectures would be valuable and informative. However, as you noted, conducting such a comparative analysis would require a significantly different approach and framing throughout the paper. Thus, after careful consideration, we followed your suggestion by expanding our investigation to include other recent OpenAI models which have not yet been systematically explored in the context of scope ambiguity.

We initially aimed to include the full o1 model and o3, as suggested. However, the full o1 model could not be run due to its extensive prompt and inference token requirements, which exceeded our resource constraints for large-scale experimentation (estimated at $15 per million input tokens and $60 per million output tokens for o1). Additionally, the full o3 model was not yet available through the OpenAI API at the time our experiments were conducted. We later noted that GPT-4.1 was released on April 14, 2025, and the full o3 model followed on April 16, 2025. However, due to time limitations, as well as continued cost constraints associated with the full o3 model (estimated at $10 per million input tokens and $40 per million output tokens), we were unable to incorporate these models into the present study. Nonetheless, we hope that the inclusion of o1-mini and o3-mini—recent, underexplored models with distinct training and alignment characteristics—meaningfully strengthens the manuscript and addresses your suggestion for broader model coverage.

Given these constraints, we included two recently released models—o1-mini (September 2024) and o3-mini (January 2025)—as accessible alternatives to assess whether our findings generalize to newer versions of the GPT architecture, which have not yet been systematically explored in the context of scope ambiguity. While these models are part of the GPT family, they differ meaningfully from earlier versions in terms of their architecture, including tuning strategies, optimization goals, and model scales. Therefore, we believe this approach enriches the manuscript by allowing us to examine how variations in training skills or model scale affect model behavior, while also offering broader implications that can inform future improvements in language model performance. We deepen the discussion by engaging in more fine-grained model comparisons through the inclusion of two newer, underexplored models.

Additionally, in the Discussion section, we briefly report findings from an exploratory evaluation using Anthropic's Claude 3.5 Sonnet, which similarly showed LA preferences across all three languages. These results are provided in the Supporting Information and included as part of our suggestions for future research.

To further reflect this expansion and strengthen the overall contribution of our paper, we have highlighted several key points in the revised manuscript:

(1) ***Abstract****: Updated to reflect the inclusion of findings from o1-mini and o3-mini to provide a more complete overview of the study's contributions (lines 9-13).*

(2) ***Introduction****: clarified the rationale for focusing on GPT models and emphasize the significance of comparing different model versions.*

(3) ***Materials and Methods****: Added detailed descriptions of the architectural and functional differences across all GPT models examined, including o1-mini and o3-mini.*

(4) ***Analysis and Results****: Integrated new results from o1-mini and o3-mini, with proportions and statistical analysis presented in Table 2.*

(5) ***Discussion****: divided into two subsections—Cross-linguistic Model Comparisons and Model Reasoning and Future Directions— to improve readability and better highlight the implications of our findings through more in-depth discussion.*

(6) ***Supporting Information****: Included the dataset and results from an exploratory evaluation using Anthropic's Claude 3.5 Sonnet, which showed similar LA preferences across languages, to support the generalizability of our findings and guide future work.*

We hope these revisions broaden the manuscript's scope while maintaining a focused and coherent contribution. The details of each revision are provided below:

1. *To clarify the rationale for investigating GPT models, we have added a statement in the Introduction highlighting their dominant role in both academic research and real-world applications (lines 20-24). We emphasized that GPT's widespread deployment makes it a natural and impactful starting point for investigating how large language models (LLMs) handle complex linguistic tasks. Additionally, we now include a statement outlining the significance of comparing multiple GPT versions—including the recent o1-mini and o3-mini models—to examine how underlying techniques and model scale shape interpretive behavior across languages (lines 82-88). This comparison, we argue, is essential for guiding the practical implications of GPT model selection and systematic strategies for enhancing multilingual capabilities.*

2. *In Materials and methods section, we now detail characteristics of each GPT model, including the recently released o1-mini and o3-mini models (lines 267-284). In particular, we highlight the inclusion of o1-mini and o3-mini as an important contribution, given their underexplored performance on complex linguistic phenomena, including structural ambiguity, and their distinct technical characteristics relative to other GPT models. We believe this addition allows for a more comprehensive analysis of interpretive behavior across different stages of GPT development.*

3. *The results of o1-mini and o3-mini are now included in Table 2 (pp. 15-17) and integrated into the Analysis and Results section (lines 318-356). Notably, these models showed strong LA preferences in both Japanese and Korean—mirroring the pattern observed in English. This contrasts with earlier GPT models, in which Japanese did not exhibit a strong preference for either interpretation, and GPT-4o even showed a significant HA preference, aligning more closely with native Japanese speakers. These findings from o1-mini and o3-mini are now incorporated into the manuscript to support a deeper discussion of model behavior across different versions.*

4. *We have extended the discussion to provide a more detailed comparison of GPT models, focusing on their distinct architectures and alignment strategies. As a result of this expanded analysis, the Discussion section is now divided into two subsections—(1) Cross-linguistic Model Comparisons and (2) Model Reasoning and Future Directions—to enhance readability and clearly present these key points. This restructuring reflects our deeper exploration of GPT model comparisons and allows us to systematically examine how differences in model design, scale, and training influence interpretive behavior across languages. These detailed comparisons are now included in the Cross-linguistic Model Comparisons subsection (lines 391-394; lines 403-431).*

   Additionally, in the Model Reasoning and Future Directions subsection, we

briefly report the results of an exploratory evaluation using Anthropic's Claude 3.5 Sonnet, which showed a similar pattern of strong LA preferences across all three languages. Since it goes beyond the scope of the current study, these findings are provided in the Supporting Information to support our discussion of broader interpretive tendencies across LLMs and to inform directions for future cross-architecture comparisons (lines 477-488).
